# IgG4-related mastitis requiring differentiation from breast cancer: a case report

**DOI:** 10.1093/jscr/rjaa240

**Published:** 2021-11-29

**Authors:** Yuka Asano, Shinichiro Kashiwagi, Yuko Kawano, Sayaka Tanaka, Yuko Kuwae, Tsutomu Takashima, Masahiko Ohsawa, Kosei Hirakawa, Masaichi Ohira

**Affiliations:** Department of Breast and Endocrine Surgery, Osaka City University Graduate School of Medicine, Osaka 545-8585, Japan; Department of Breast and Endocrine Surgery, Osaka City University Graduate School of Medicine, Osaka 545-8585, Japan; Department of Breast and Endocrine Surgery, Osaka City University Graduate School of Medicine, Osaka 545-8585, Japan; Department of Diagnostic Pathology, Osaka City University Graduate School of Medicine, Osaka 545-8585, Japan; Department of Diagnostic Pathology, Osaka City University Graduate School of Medicine, Osaka 545-8585, Japan; Department of Breast and Endocrine Surgery, Osaka City University Graduate School of Medicine, Osaka 545-8585, Japan; Department of Diagnostic Pathology, Osaka City University Graduate School of Medicine, Osaka 545-8585, Japan; Department of Breast and Endocrine Surgery, Osaka City University Graduate School of Medicine, Osaka 545-8585, Japan; Department of Breast and Endocrine Surgery, Osaka City University Graduate School of Medicine, Osaka 545-8585, Japan

## Abstract

Immunoglobulin (Ig) G4-related disease (IgG4-RD) is a group of chronic relapsing inflammatory conditions. Although IgG4-RD can occur in various organs, it is rarely observed in mammary glands. Here, we report a case of IgG4-related mastitis (IgG4-RM) that needed to be differentiated from breast cancer. A 54-year-old woman was examined for a tumor in her left breast. Mammary ultrasonography revealed an irregular hypoechoic tumor measuring 45.0 × 43.0 × 32.0 mm in size. A core-needle biopsy of the left breast tissue revealed a high degree of mixed T and B lymphocytic and plasma cell infiltration, as well as interstitial fibrosis. IgG4-RD was diagnosed based on hematological examination that revealed an abnormal IgG4 value of 332 mg/dl. All the clinical diagnostic criteria for IgG4 were met, resulting in a definitive diagnosis of IgG4-RM.

## INTRODUCTION

Immunoglobulin (Ig) G4-related disease (IgG4-RD) is a group of chronic inflammatory conditions [1, 2] and includes high-density lymphoplasmacytic cell invasion, fibrosis and obliterative phlebitis. Although IgG4-RD may exhibit characteristics of a systemic disease, it often remains restricted to a single organ [2]. IgG4-RD has an unknown etiology, and although it can occur in various organs, it is rarely observed in mammary glands [3]. Here, we report a case of IgG4-related mastitis (IgG4-RM) that needed to be differentiated from breast cancer.

## CASE REPORT

**Figure 1:**
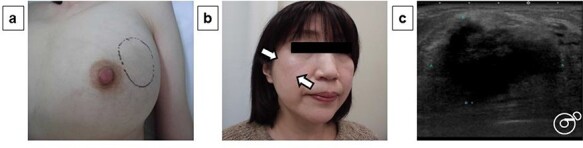
Physical examination and ultrasonography findings: During physical examination, an elastic hard mass measuring ~6 cm in size was palpated in the upper-outer area of the left breast (**a**). An induration of ~4 cm was also found on the right cheek (**b**). Mammary ultrasonography revealed an irregular hypoechoic tumor of measuring 45.0 × 43.0 × 32.0 mm in size in the upper-outer quadrant of the left breast (**c**).

**Figure 2:**
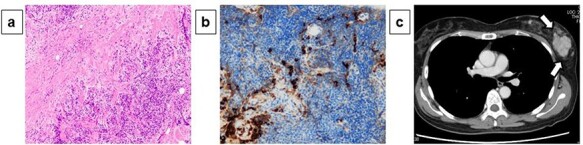
Pathological diagnosis of core-needle biopsy specimens and computed tomography findings: Core-needle biopsy performed in the same site revealed a high degree of mixed T and B lymphocytic and plasma cell infiltration, as well as interstitial fibrosis (×200) (**a**). Many plasma cells were IgG positive, of which >40% were IgG4 positive (×200) (**b**). Computed tomography exhibited no significant findings besides the left mammary mass and left axillary swelling (**c**).

A 54-year-old woman presented with a tumor in her left breast. The patient had no relevant medical or family history. During physical examination, an elastic hard mass measuring ~6 cm in size was palpated in the upper-outer area of the left breast (Fig. 1a). An induration of ~4 cm was also found on the right cheek (Fig. 1b). Mammary ultrasonography revealed an irregular hypoechoic tumor of measuring 45.0 × 43.0 × 32.0 mm in size in the upper-outer quadrant of the left breast (Fig. 1c). Core-needle biopsy performed in the same site revealed a high degree of mixed T and B lymphocytic and plasma cell infiltration, as well as interstitial fibrosis (Fig. 2a). Many plasma cells were IgG positive, of which >40% were IgG4 positive (Fig. 2b). These biopsy findings led to a diagnosis of IgG4-RM of the left breast. Computed tomography exhibited no significant findings besides the left mammary mass and left axillary swelling (Fig. 2c). IgG4-RD was diagnosed based on hematological examination results revealing elevated serum IgG4 levels (332 mg/dl). Based on comprehensive diagnostic criteria for IgG4-RDs, we diagnosed the patient with IgG4-RM. The condition was treated with prednisolone (30 mg/day, 0.6 mg/kg/day) for 4 weeks, which resulted in a decrease in the tumor size. The patient is currently being followed up with maintenance therapy.

## DISCUSSION

IgG4-RD represents a group of multisystem, mass-forming entities with increased serum and tissue IgG4 levels and characteristic histomorphological findings such as dense lymphoplasmacytic infiltration, storiform fibrosis and obliterative phlebitis [1, 2, 4–6]. The existing comprehensive diagnostic criteria for IgG4-RD include the following items [2]: (i) clinical findings such as diffused or localized enlargement, mass, nodular and/or hypertrophic lesions in single or multiple organs; (ii) hematological findings such as high IgG4emia (≥135 mg/dl) and (iii) pathological findings such as prominent lymphocytes, plasma cell infiltration and fibrosis, with an IgG4-positive/IgG-positive cell ratio ≥40% and >10 IgG4-positive plasma cells per high-powered microscopic field. In our case, all the aforementioned criteria were met, and the patient was thus diagnosed as having IgG4-RM; the mammary gland and axillary lymph node findings were in accordance with the clinical criteria, the serum IgG4 level met the hematological criterion (332 mg/dl; reference range 11–121 mg/dl) and the histological findings of the mammary gland tissue and lymph node met the pathological criteria (a high degree of mixed T and B lymphocytic and plasma cell infiltration and an IgG4-positive/IgG-positive cell ratio >40%).

IgG-RD is frequently observed in the pancreas, lacrimal gland and salivary gland, whereas reports of IgG-RD affecting the mammary gland are rare [3, 7]. The current hypothesis of IgG4-RD immune disorders is a B-cell-mediated immunologic response to an unknown antigen, with follicular T-helper cells modulating a class switch toward IgG4 [8]. Estrogen can act as immunomodulators on immune cells, which have been extensively studied to understand the prevalence of many autoimmune disorders in women [9]. In other words, estrogen may play a role in some patients with IgG4-RM. In previous reports, IgG4-RM was mostly found in middle-aged women, and its location was no different between left and right, indicating a favorable prognosis [3, 7].

Steroid therapy is considered the first-choice treatment for IgG4-RD [10]. The recommended course of treatment is 0.6 mg/kg/day for 2–4 weeks, followed by 5 mg/day for 3–6 months, and maintenance therapy at 2.5–5 mg/day for 3 years. In previous reports of IgG4-RM, radical surgery was also performed; however, it appears to be an excessive procedure with respect to the effectiveness of steroid therapy [7].

In conclusion, we have reported a rare case of IgG4-RM requiring differentiation from breast cancer alongside a discussion of the literature.
